# Estimating the impact of a cancer diagnosis on life expectancy by socio-economic group for a range of cancer types in England

**DOI:** 10.1038/bjc.2017.300

**Published:** 2017-09-12

**Authors:** Elisavet Syriopoulou, Hannah Bower, Therese M-L Andersson, Paul C Lambert, Mark J Rutherford

**Affiliations:** 1 Biostatistics Research Group, Department of Health Sciences, University of Leicester, Centre for Medicine, University Road, Leicester LE1 7RH, UK; 2 Department of Medical Epidemiology and Biostatistics, Karolinska Institutet, SE-171 77, Stockholm, Sweden

**Keywords:** socio-economic differences, life expectancy, proportion of life lost, total number of years lost

## Abstract

**Background:**

Differences in cancer survival exist across socio-economic groups for many cancer types. Standard metrics fail to show the overall impact for patients and the population.

**Methods:**

The available data consist of a population of ∼2.5 million patients and include all patients recorded as being diagnosed with melanoma, prostate, bladder, breast, colon, rectum, lung, ovarian and stomach cancers in England between 1998 and 2013. We estimated the average loss in expectation of life per patient in years and the proportion of life lost for a range of cancer types, separately by deprivation group. In addition, estimates for the total number of years lost due to each cancer were also obtained.

**Results:**

Lung and stomach cancers result in the highest overall loss for males and females in all deprivation groups in terms of both absolute life years lost and loss as a proportion of expected life remaining. Female lung cancer patients in the least- and most-deprived group lose 14.4 and 13.8 years on average, respectively, that is translated as 86.1% and 87.3% of their average expected life years remaining. Melanoma, prostate and breast cancers have the lowest overall loss. On the basis of the number of patients diagnosed in 2013, lung cancer results in the most life years lost in total followed by breast cancer. Melanoma and bladder cancer account for the lowest total life years lost.

**Conclusions:**

There are wide differences in the impact of cancer on life expectancy across deprivation groups, and for most cancers the most affluent lose less years.

In many countries cancer survival varies by socio-economic status (Dalton *et al*, 2008; Van der Heyden *et al*, 2009; Sprague *et al*, 2011; Coleman, 2014; Ito *et al*, 2014). In the United Kingdom there have been national policies aimed at reducing inequalities (Department of Health, 2000; Department of Health, 2011), but any changes have been minor (Rachet *et al*, 2009; Rachet *et al*, 2010). Part of the observed differences in survival between socio-economic groups can be explained by stage at diagnosis (Rutherford *et al*, 2013; Morris *et al*, 2016).

Approaches to estimate the impact of cancer for a patient tend to rely on metrics that are relevant at a particular point in follow-up time after diagnosis, such as 1- or 5-year relative survival (Andersson *et al*, 2013). Relative survival is a measure that is not influenced by mortality due to other causes and is useful for comparing cancer survival between population groups or across time. However, it does not provide information on the life-time impact of a diagnosis of cancer.

An alternative measure that looks over the whole of the remaining life time is the loss in expectation of life resulting from a diagnosis of cancer (Andersson *et al*, 2015). Loss in expectation of life due to cancer is the difference between the life expectancy of those who are not diagnosed with cancer and the life expectancy of patients diagnosed with cancer (Andersson *et al*, 2013). It is possible to estimate a patient’s loss in expectation of life based on the individual’s characteristics such as age, sex and stage at diagnosis. To understand the impact of a cancer at a population level, the average and total loss in expectation of life for subgroups, for example, certain deprivation groups or stage groups, can be obtained. Compared to 5-year relative survival, loss in expectation of life is a more intuitive measure that can be easily interpreted and estimates the impact of the cancer for the whole lifespan of a person (Rutherford *et al*, 2015; Bower *et al*, 2016).

We assess the life-time impact of a diagnosis of cancer and how this varies by socio-economic status for a range of cancer types in England by estimating the loss in expectation of life and proportion of life lost.

## Materials and methods

### Data resources

Data were obtained on all individuals in England diagnosed with one of the cancer types of interest between the start of 1998 and the end of 2013 using National Cancer Registry Data provided by Public Health England. Cancer types with varying prognosis and characteristics such as age at diagnosis were carefully selected. The analysis included lung, stomach, ovarian, bladder, colon, rectum and breast cancers, melanoma and prostate cancer. International Classification of Diseases 10 was used to identify individuals with these cancers (see Table 1 for details). Information on the socio-economic status of the patients was available, and each of the patients was classified to one of five deprivation groups. The categorisation was based on national quintiles of the income domain of the index of multiple deprivation (IMD) 2010 score of the lower super output area of patients’ residence at diagnosis (Neighbourhood Renewal Unit, 2004; Department for Communities and Local Government, 2011) and was available for the whole population of England. The overall IMD is a weighted area-level aggregation and not an individual-specific measure. For patients with multiple tumours only the first tumour for each cancer type was considered.

### Statistical methods

We fitted flexible parametric relative survival models that use restricted cubic splines to capture the shape of the baseline excess hazard (Royston, 2001; Royston and Parmar, 2002; Nelson *et al*, 2007). Separate models were fitted for each cancer type and sex. From these models we predicted the loss in expectation of life and the proportion of life lost for each of the five deprivation groups using the approach by Andersson *et al*, 2013. Andersson *et al* showed that it is possible to consistently extrapolate cancer survival by extrapolating relative survival rather than observed survival. The main intuition of this approach is that as time since diagnosis increases the expected mortality rate dominates. Expected mortality rates were obtained from population mortality files stratified by sex, age, deprivation group and calendar year (Spika *et al*).

The models included deprivation group and age at diagnosis. Age was included in the models as a continuous variable but it was allowed to be non-linear through use of restricted cubic splines. The effect of deprivation and age at diagnosis were assumed to be time-dependent (i.e. relaxing the proportional excess hazards assumption). An interaction between age and deprivation was also included.

A period analysis with a period window between years 2007 and 2013 was conducted. The analysis included all individuals who were under follow-up at any point from the beginning of the year 2007 until 31 December 2013, independent of whether they were diagnosed before or after year 2007. Period analysis has been widely shown to provide good predictions of the prognosis of newly diagnosed patients and highlights temporal trends in patient survival sooner than cohort methods (Brenner *et al*, 2002; Brenner and Hakulinen, 2007).

We calculated the averaged loss in expectation of life for each cancer type by a weighted average of the age-specific estimates. To do so we first estimate age-specific estimates within each deprivation, cancer type and sex group, and then obtain the weighted average. The weights reflect the age distribution of those diagnosed in year 2013, the most recent year in our study population for each deprivation group separately (internal standardisation). Loss in expectation of life is a highly age-dependent measure because those at younger ages have more life years to lose initially. We therefore also estimated the proportion of life lost due to cancer. The proportion of life lost is defined as the loss in expectation of life for a person in our cancer population divided by the expectation of life for those who are not diagnosed with cancer but with similar characteristics.

We also estimated the total life years that would be lost due to cancer for a typical annual cohort size in England, using the cohort diagnosed in the year 2013 for each cancer type. This measure is calculated by multiplying the number of patients diagnosed with cancer by the average loss in expectation of life for each deprivation group.

All statistical analyses were conducted using Stata 14.1 (StataCorp., 2015).

See Supplementary Material for further details.

## Results

A population of ∼2.5 million cancer patients diagnosed with one of the nine cancer types were included in our analysis. Table 1 shows the number of patients included for each type of cancer, as well as the mean age of the patients by deprivation group. On average melanoma patients are diagnosed at the youngest age, whereas bladder cancer patients have the oldest age at diagnosis. Age at diagnosis varies slightly by deprivation group for all the cancer types. There was notable variation in the number of patients diagnosed in each deprivation group for melanoma, prostate, breast and lung cancers.

**Figure 1:**
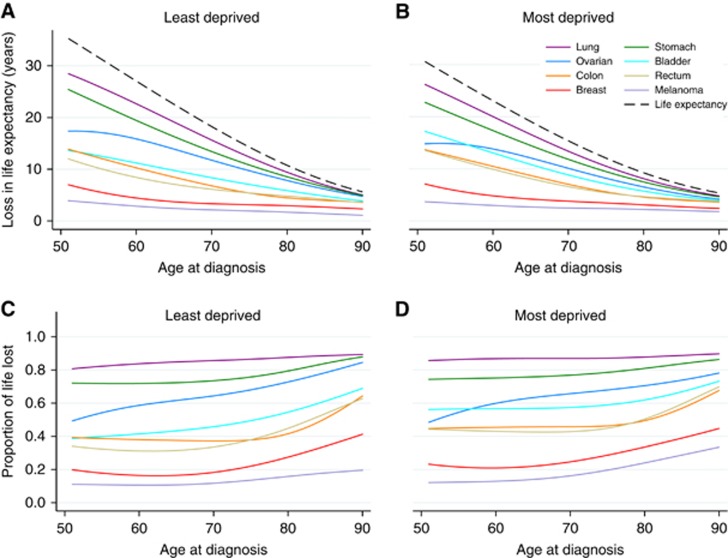
**Estimates from a period analysis 2007–2013 (England) for female cancer types.** (**A**) Average loss in expectation of life in years for the least deprived, (**B**) average loss in expectation of life in years for the most deprived, (**C**) proportion of life lost for the least deprived and (**D**) proportion of life lost for the most deprived. Dashed lines in plots **A** and **B** show the life expectancy in the general population.

Figure 1 shows the loss in expectation of life and the proportion of life lost for the least- and the most-deprived groups across age at diagnosis for different cancer types in females. The life expectancy of the general population, which is free of cancer, is given in the dashed line in Figure 1. Lung and stomach cancers have the highest loss in all deprivation groups. Melanoma and breast cancer have comparatively much lower loss in expectation of life. Estimates for loss in expectation of life vary widely by age at diagnosis for all cancers except for melanoma and breast cancer. For example, those who were diagnosed at the age of 50 with lung cancer lose nearly 30 years of remaining life, whereas patients above 80 years of age lose <10 years. The main cause of death for younger patients is their cancer but death due to other causes becomes more likely as age increases. There is less variation by age for the proportion of life lost, but the variation between the cancer types is similar. For example, females with lung cancer lose ∼87% of their lives for all ages at diagnoses. Results for males were similar and are shown in the Supplementary Figure. Table 2 shows the average loss in expectation of life and the average proportion of life lost, by standardising to the age distribution within each cancer type, deprivation group and sex in 2013. Women lose more years than men, except for melanoma. The highest average loss in expectation of life is for female lung cancer patients at 14.4 to 13.8 years for the least and most deprived, respectively. High values are also observed for males with lung cancer, stomach cancer for both males and females and patients with ovarian cancer. The lowest loss in expectation of life was for females with melanoma; the average loss was 2.9 and 3.1 for the least and most deprived, respectively. Low averages are also observed for prostate and breast cancers. For most of the cancers the loss in expectation of life increases from the least- to the most-deprived groups. A decreasing trend across deprivation groups is observed for prostate, lung, stomach and ovarian cancers.

For some cancer types the decreasing trend in loss in expectation of life across deprivation groups is reversed when considering the proportional scale (e.g., stomach cancer, lung cancer and males with bladder cancer). For example, females with lung cancer lose 14.4 and 13.8 years on average for the least and most deprived, respectively. The equivalent average percentages of life lost are 86.1% and 87.3%, respectively. This is because mortality due to other causes in the more-deprived group is higher. A decreasing trend from least to most deprived in proportion of life lost was also found for ovarian cancer. On the proportional scale, the highest loss is for males with lung cancer and varies from 87.6 to 88.1% of expected remaining life lost. The lowest loss is for females with melanoma and varies from 11.7 to 14.3% across deprivation groups.

The average loss in expectation of life estimates were similar for ovarian cancer and stomach cancer for female patients, ranging between 10.6 and 11.4 years. However, on the proportion of life lost scale the estimates differ due to the higher average age at diagnosis for stomach cancer. For stomach cancer, the proportion of life lost remains above 77% for all deprivation groups while for ovarian cancer the highest estimate is that of the least deprived at ∼60%.

**Figure 2:**
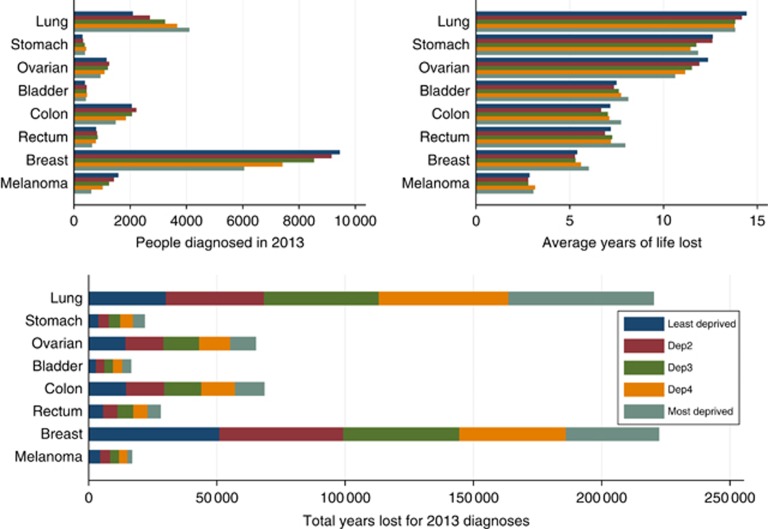
**Total years lost in England due to cancer diagnosis in 2013 for females (bottom plot) as a product of the number of patients diagnosed in 2013 (top left plot) and average loss in expectation of life (top right plot) by cancer type and deprivation group.** Estimates were obtained from a period analysis 2007–2013.

**Figure 3:**
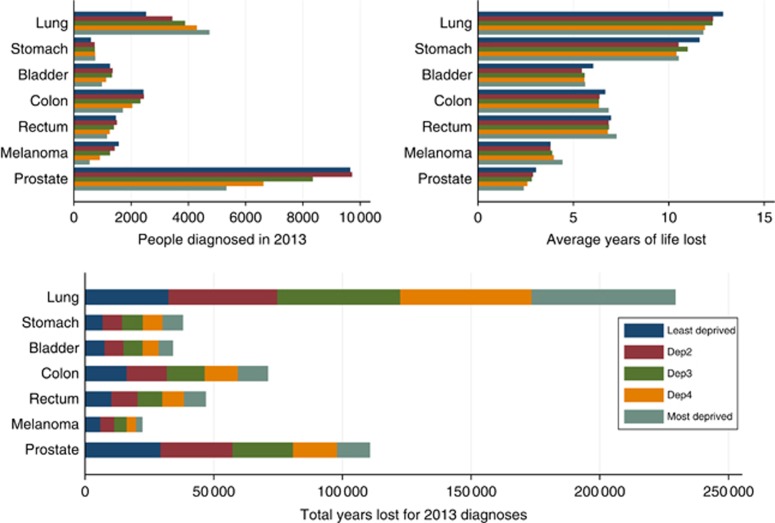
**Total years lost in England due to cancer diagnosis in 2013 for males (bottom plot) as a product of the number of patients diagnosed in 2013 (top left plot) and average loss in expectation of life (top right plot) by cancer type and deprivation group.** Estimates were obtained from a period analysis 2007–2013.

Figures 2 and 3 and Table 3 show the number of patients diagnosed, the estimated total number of life years lost and the mean years lost by sex, deprivation group and cancer type in 2013. Lung cancer results in the largest total life years lost, with the number of years lost in 2013 diagnoses above 30 000 years for all deprivation groups and both genders. The total life years lost estimates are influenced by both the number of patients diagnosed and the average years of life lost. For example, even though breast cancer results in few years of life lost on average, because there are many women diagnosed with the cancer, breast cancer has the second largest total life years lost. Prostate cancer has also low average years of life lost but it has large total years lost due to its higher incidence. Melanoma and bladder cancer have the lowest total life years lost. Males lose more years in total in comparison with females for all the cancers considered.

## Discussion

We have estimated the impact of cancer diagnosis by socio-economic group using absolute and proportional measures of loss in expectation of life for a range of cancer types in England. Our results showed that lung and stomach cancers have the highest loss in expectation of life, whereas melanoma, prostate and breast cancers have the lowest loss on average. A similar pattern was found for the proportion of life lost due to cancer. For some cancers (e.g., lung and stomach cancers) we observed a decreasing effect on loss in expectation of life across deprivation groups, but the trend was reversed for the proportion of life lost. For lung cancer the loss corresponds to nearly all of patients’ lives as the proportion of life lost is higher than 85% for all groups. As the least-deprived patients in the general population have a higher life expectancy to begin with they have more years to lose. Thus, loss in expectation of life due to cancer will be higher in the least-deprived group. The reversed trend in proportion of life lost is the result of different background mortalities between the deprivation groups. For ovarian cancer, the proportion of life lost remains decreasing with increasing deprivation. This could be partially explained by small differences in cancer-related survival (similar relative survival) and larger different differences in the expected background survival of the general population across deprivation groups. Moreover, the most-deprived group has a younger population in comparison to the least-deprived group. Lung cancer results in the largest total life years lost followed by breast cancer even though it affects only females and not the whole population. Bladder and melanoma cancers result in the lowest total life years lost.

Our results are consistent with previous studies that suggest that survival after a cancer diagnosis in England is markedly different across socio-economic groups with the most deprived usually having lower survival (Coleman *et al*, 2004; Rachet *et al*, 2008), even when adjusting for differences in background survival. We, however, report these differences using a metric that allows easier communication of the impact of cancer on life expectancy by taking a view across the entire lifetime of individuals diagnosed with cancer. Caution is needed when trying to interpret our results in terms of how well the health-care system works for cancer in England. In the general population, which is free of the cancer of interest, the least-deprived group will have a higher life expectancy meaning more life years to lose to begin with. This might result in higher loss in expectation of life for the most affluent. An evaluation of the 2000 NHS Cancer Plan (Department of Health, 2000; Rachet *et al*, 2010), which aimed to improve survival and reduce inequalities, showed that even though survival after cancer diagnosis has improved in recent years, disparities in cancer outcomes between the least- and the most-deprived groups continue to persist. Similar inequalities in survival have also been reported in other countries in Europe or elsewhere (Dalton *et al*, 2008; Siemerink *et al*, 2011; Singh *et al*, 2011). Even though the tumour biology, patients’ characteristics, such as comorbidity, health-seeking behaviours and psychosocial factors, and screening could explain part of the observed differences, the underlying factors for the gap among deprivation groups remain controversial (Kaffashian *et al*, 2003; Lyratzopoulos *et al*, 2003; Morris *et al*, 2015). Stage at diagnosis and differences in treatment have been considered to be key factors for differences in survival (Woods *et al*, 2006; Rutherford *et al*, 2013). Even though it is anticipated that the estimates in our analysis will vary significantly by stage at diagnosis, no conclusions can be made for the actual variation as information on stage is not included in the analysis. Moreover, like all survival measures, loss in expectation of life can potentially be influenced by screening. However, it is still a useful summary to estimate life expectancy at the population level, including all individuals diagnosed through screening or symptomatically. We consider the differences due to screening to be negligible. The explanations for socio-economic differences is not well documented and further research is required to improve understanding of the factors that drive differences in survival.

To estimate loss in expectation of life using our approach, extrapolation of relative survival is necessary. This has previously been shown to be robust (Andersson *et al*, 2013), but we performed a sensitivity analysis with regard to some of the assumptions made when extrapolating to ensure that the estimates are not overly influenced (see Supplementary Material for details). Period analysis was also used so that the most recently available information on cancer patient survival is used. In this way, patients with the longest follow-up are used for the long-term predictions and newly diagnosed patients are used for the short-term survival meaning that long extrapolation is not required (Brenner and Hakulinen, 2006; Brenner and Hakulinen, 2009). The use of period analysis strengthens the analysis as we can get reliable estimates of long-term survival.

Loss in expectation of life, both on the absolute and proportional scale, as well as the total life years lost measures are not directly comparable across deprivation groups as the age distribution may vary across groups. The main interest of the study was to calculate estimates that were relevant to the English population and its characteristics (such as age at diagnosis) rather than an international standard population (Corazziari *et al*, 2004) that would allow a more fair comparison but has different characteristics. This enables the identification the least- and most-affected groups in England. To ensure that average estimates are not influenced by imbalances in our population (particularly by age) we also conducted an external standardisation additionally to the internal standardisation. External standardisation, for the average estimates of loss in expectation of life and proportion of life lost in Table 2, was performed by either calculating weights from the patients diagnosed in year 2013 in all deprivation groups or by using the International Cancer Survival Standard weights. Different ways of averaging did not have an important influence on the comparisons between deprivation groups as there were not large difference in age between the deprivation groups.

Loss in expectation of life is a measure that is highly dependent on age. Younger patients have more years to lose than older patients and this heavily affects the loss in expectation of life measure. The proportion of life lost is a more stable measure across age and can be used to report the impact of cancer on patients’ life expectancy. However, it is not a perfect measure for performing comparisons across groups with differing background survival and it is still influenced by the expected life remaining; if two groups have the same relative survival, but different expected survival, the proportional measure will result in different estimates. However, it does provide a ‘real world’ measure of survival. Both loss in expectation of life and proportion of life can improve communication and provide further understanding on how inequalities, such as socio-demographic differences, affect cancer patient survival across their whole lifetime and they can be a measure of great interest for public health, clinicians and potentially patients. They can also be used to quantify the disease burden for society, drive policy interventions and motivate awareness campaigns for the most-affected subgroups to eliminate inequalities whenever this is possible. The measures are easy to interpret and existing software can be used for their estimation. Together with relative survival measures, which are more appropriate when the interest is in comparisons across groups whilst removing different expected mortality rates, the measures presented here can help us understand different aspects of the cancer of interest. On the one hand, relative survival is useful for making comparisons across time, across different age groups in our population and across different countries. On the other hand, loss in expectation of life provides ‘real world’ estimates for the actual impact of cancer in the population of interest.

Even though survival after a cancer diagnosis has increased during the last years, the impact of cancer on life expectancy varies across socio-economic groups. The gap between the most and least affluent suggest that further action is required to tackle health inequalities, by ensuring access to screening procedures and optimal health-care services and treatment for the whole population. Further statistical analysis should focus on the underlying determinants of these inequalities. Loss in expectation of life measures and total life years lost are easily understood measures that can be used to improve understanding and explore variation therefore their use is encouraged.

## Figures and Tables

**Table 1:** Number of patients (mean age at diagnosis) for different cancer types by sex and deprivation group in England

	**Deprivation quintile**					
**Cancer type**	**Least deprived**	**2**	**3**	**4**	**Most deprived**	***N*** **(age) total**
**Lung**						
Males	39 491 (72.1)	50 988 (72.1)	58 584 (71.8)	66 698 (71.2)	75 653 (70.1)	291 414 (71.3)
Females	27 208 (71.8)	35 866 (71.9)	42 039 (71.9)	49 477 (71.6)	57 420 (70.7)	212 010 (71.5)
**Stomach**						
Males	10 741 (71.5)	12 954 (72.0)	13 883 (72.0)	14 737 (71.8)	15 472 (70.9)	67 787 (71.6)
Females	5337 (73.8)	6579 (74.7)	7573 (74.9)	8217 (74.7)	8778 (73.8)	36 484 (74.4)
**Ovarian**						
Females	17 688 (64.1)	19 466 (64.5)	19 173 (65.0)	17 540 (64.1)	14 960 (62.3)	88 827 (64.0)
**Bladder**						
Males	19 171 (73.1)	21 561 (73.4)	21 714 (73.3)	20 530 (72.8)	17 845 (71.7)	100 821 (72.9)
Females	6621 (75.0)	7976 (75.5)	8214 (75.6)	8527 (75.3)	7683 (74.3)	39 021 (75.1)
**Colon**						
Males	32 079 (70.7)	34 187 (71.2)	32 618 (71.3)	29 593 (71.1)	25 855 (70.2)	154 332 (70.9)
Females	28 687 (72.0)	32 140 (73.0)	31 885 (73.3)	29 186 (73.3)	24 167 (72.4)	146 065 (72.8)
**Rectum**						
Males	21 125 (68.6)	22 542 (69.2)	22 411 (69.4)	20 858 (69.3)	19 029 (68.6)	105 966 (69.0)
Females	12 995 (70.1)	14 500 (71.0)	14 403 (71.5)	13 414 (71.6)	11 484 (70.7)	66 796 (71.0)
**Breast**						
Females	128 807 (61.5)	131 004 (62.7)	123 949 (63.3)	109 975 (63.3)	89 758 (62.5)	583 493 (62.7)
**Melanoma**						
Males	16 822 (61.8)	15 542 (62.3)	13 257 (62.1)	9820 (61.2)	6156 (59.8)	61 597 (61.7)
Females	18 299 (58.2)	17 459 (59.2)	15 097 (59.3)	11 886 (58.5)	7533 (56.6)	70 274 (58.6)
**Prostate**						
Males	113 190 (71.0)	115 485 (71.6)	103 140 (72.0)	86 737 (72.0)	69 306 (71.7)	487 858 (71.6)

International Classification of Diseases 9 and 10: lung cancer (ICD10: C34); stomach cancer (ICD10: C16); ovarian cancer (ICD10: C56); bladder cancer (ICD10: C67); colon cancer (ICD10: C18); rectum cancer (ICD10: C19 and C20); breast cancer (ICD10: C50); melanoma (ICD10: C43); prostate cancer (ICD10: C61).

**Table 2:** Average loss in expectation of life and proportion of life lost for each deprivation group and various cancer types by sex in England

	**Average loss in life expectancy (years)**					**Average percentage of life lost (%)**				
**Cancer type**	**Least deprived**	**2**	**3**	**4**	**Most deprived**	**Least deprived**	**2**	**3**	**4**	**Most deprived**
**Lung**										
Males	12.84	12.31	12.29	11.89	11.81	87.55	87.96	88.19	88.20	88.12
Females	14.42	14.17	13.82	13.78	13.82	86.07	86.75	86.75	87.03	87.26
**Stomach**										
Males	11.60	10.51	10.99	10.40	10.52	78.27	78.32	78.58	79.29	79.53
Females	12.63	12.59	11.75	11.44	11.84	77.07	78.98	79.23	78.17	79.00
**Ovarian**										
Females	12.37	11.91	11.51	11.15	10.62	60.32	58.79	59.23	57.17	54.15
**Bladder**										
Males	6.04	5.44	5.58	5.58	5.61	45.42	44.30	45.69	47.09	48.26
Females	7.49	7.35	7.61	7.73	8.11	51.17	53.35	54.19	58.68	60.89
**Colon**										
Males	6.67	6.37	6.34	6.34	6.84	43.21	43.38	44.17	45.17	48.18
Females	7.15	6.67	7.02	7.10	7.73	41.71	42.40	43.95	44.94	48.91
**Rectum**										
Males	6.97	6.84	6.86	6.79	7.26	40.55	41.88	43.24	45.16	47.77
Females	7.18	6.88	7.25	7.19	7.96	38.95	39.01	43.58	43.54	47.61
**Breast**										
Females	5.39	5.28	5.30	5.60	6.01	21.54	22.35	22.84	24.61	26.82
**Melanoma**										
Males	3.79	3.79	3.88	3.97	4.42	19.66	20.14	21.36	21.28	23.48
Females	2.85	2.78	2.78	3.15	3.06	11.73	12.47	12.37	14.30	14.05
**Prostate**										
Males	3.04	2.88	2.81	2.58	2.39	21.12	21.05	21.50	20.99	20.07

Estimates were obtained from a period analysis 2007–2013. Average estimates were calculated after internal weights were used.

**Table 3:** Total life years lost due to cancer diagnosis in 2013 in England by cancer type, deprivation group and sex

		**Males**			**Females**		
**Cancer type**	**Deprivation group**	**Group size in 2013**	**Mean years life lost**	**Total life years lost**	**Group size in 2013**	**Mean years life lost**	**Total life years lost**
Lung	Least deprived	2525	12.84	32 416	2088	14.42	30 112
	2	3437	12.31	42 315	2700	14.17	38 268
	3	3883	12.29	47 728	3238	13.82	44 750
	4	4292	11.89	51 042	3668	13.78	50 538
	Most deprived	4737	11.81	55 952	4100	13.82	56 648
Stomach	Least deprived	593	11.60	6882	305	12.63	3852
	2	720	10.51	7567	323	12.59	4068
	3	729	10.99	8012	378	11.75	4442
	4	741	10.40	7705	432	11.44	4942
	Most deprived	746	10.52	7848	387	11.84	4583
Ovarian	Least deprived				1161	12.37	14 356
	2				1248	11.91	14 864
	3				1208	11.51	13 906
	4				1083	11.15	12 073
	Most deprived				945	10.62	10 035
Bladder	Least deprived	1260	6.04	7605	386	7.49	2892
	2	1348	5.44	7330	448	7.35	3294
	3	1332	5.58	7438	442	7.61	3364
	4	1123	5.58	6262	466	7.73	3604
	Most deprived	975	5.61	5466	424	8.11	3439
Colon	Least deprived	2430	6.67	16 205	2053	7.15	14 683
	2	2443	6.37	15 553	2217	6.67	14 783
	3	2324	6.34	14 738	2065	7.02	14 501
	4	2039	6.34	12 929	1845	7.10	13 102
	Most deprived	1708	6.84	11 687	1483	7.73	11 467
Rectum	Least deprived	1464	6.97	10 200	790	7.18	5673
	2	1499	6.84	10 256	816	6.88	5612
	3	1395	6.86	9568	843	7.25	6114
	4	1245	6.79	8456	779	7.19	5601
	Most deprived	1158	7.26	8402	642	7.96	5110
Breast	Least deprived				9452	5.39	50 981
	2				9154	5.28	48 303
	3				8529	5.30	45 226
	4				7410	5.60	41 494
	Most deprived				6054	6.01	36 394
Melanoma	Least deprived	1564	3.79	5929	1578	2.85	4500
	2	1425	3.79	5400	1423	2.78	3953
	3	1263	3.88	4906	1243	2.78	3460
	4	908	3.97	3603	1018	3.15	3206
	Most deprived	547	4.42	2418	617	3.06	1887
Prostate	Least deprived	9651	3.04	29 335			
	2	9716	2.88	28 003			
	3	8343	2.81	23 484			
	4	6620	2.58	17 111			
	Most deprived	5326	2.39	12 725			

Estimates were obtained from a period analysis 2007–2013.
