# Supplementary material for: Estimating the impact of a cancer diagnosis on life expectancy by socio-economic group for a range of cancer types in England
Source: Br J Cancer. 2017 Sep 12;117(9):1419–26. doi: 10.1038/bjc.2017.300 (PMC5672926; doi:10.1038/bjc.2017.300)
Supplement: Supplementary Information [file bjc2017300x1.docx]

Supplementary material

We fitted flexible parametric relative survival models to capture the shape of the baseline excess hazard through the use of restricted cubic splines. Restricted cubic splines are flexible mathematical functions defined by piecewise polynomials that are forced to be linear before the first and after the final points (i.e. knots) at which the polynomials join.

In relative survival framework, to incorporate the background risk of death due to other causes and estimate the expected survival, the use of lifetable data is required. In our example, the lifetable data were stratified by sex, age and deprivation-group.

In a flexible parametric model we need to specify the number of splines which are defined as 1 plus the degrees of freedom. In our models five degrees of freedom were used to model the baseline excess hazard. Age was included in the model as a continuous variable but it was allowed to be non-linear by using splines (3 degrees of freedom). Time-dependent effects for age at diagnosis and deprivation status were modelled with five degrees of freedom (except for lung cancer for males and females, bladder cancer for females and melanoma for females in which 3, 2, 3 and 3 degrees were used respectively). An interaction between age and deprivation was also considered.

Loss in expectation of life both on absolute and the proportional scale were estimated. Loss in expectation of life is given as: $LEL\left( z \right)=\int_{0}^{t_{max}} S^{*}\left( t,z^{'} \right)dt- \int_{0}^{t_{max}} S\left( t,z \right)dt,$ where $S^{*}\left( t,z^{'} \right)$ refers to the expected survival of the general population, $S(t,z)$ refers to the observed survival in the population of patients with the cancer of interest, $t_{max}$ is the assumed time at which both survival functions become zero, *z* are the covariates included in the model and $z^{'}$ is a subset of the covariates included in the population life table. The formula can be rewritten as: $LEL\left( z \right)=\int_{0}^{t_{max}} S^{*}\left( t,z^{'} \right)dt- \int_{0}^{t_{max}} S^{*}\left( t,z^{'} \right)\times R\left( t,z \right)dt,$ where $R\left( t,z \right)$ is the relative survival. Loss in expectation of life measures can be measures of great interest for public health officials, clinicians and patients as it can be used to quantify the disease burden in society and to address various research questions concerning the impact of cancer for groups of interest.

To estimate measures of loss in expectation of life it is necessary to extrapolate in the future. The approach of Andersson et al. was extended to incorporate further covariate effects and appropriate constraints were applied to ensure the extrapolated effect estimates are reasonable. All time-dependent excess hazard ratios for the effect of deprivation were constrained to be proportional beyond a given point in follow-up time (12 years for all cancers except melanoma and bladder cancers for which 10 years were used) to ensure that we do not extrapolate a misleading protective effect by allowing the hazard ratios to continue to diminish. Different split points, e.g 10 and 15 years, were considered but they did not influence the estimates.

We calculated summary estimates for each cancer site by averaging the age-specific estimates using internal standardisation. Alternative ways of standardisation were also considered. In external standardisation the weights were derived from the patients diagnosed in year 2013 as a total, meaning all deprivation groups. Furthermore, the International Cancer Survival Standard (ICSS) weights were used. ICSS weights are weights obtaining from a standard population. Average estimates are also calculated for the proportion of life lost. The different ways of standardisation did not affect the estimates.
