# Supplementary figures and images for: Estimating the impact of a cancer diagnosis on life expectancy by socio-economic group for a range of cancer types in England
Source: Br J Cancer. 2017 Sep 12;117(9):1419–26. doi: 10.1038/bjc.2017.300 (PMC5672926; doi:10.1038/bjc.2017.300)

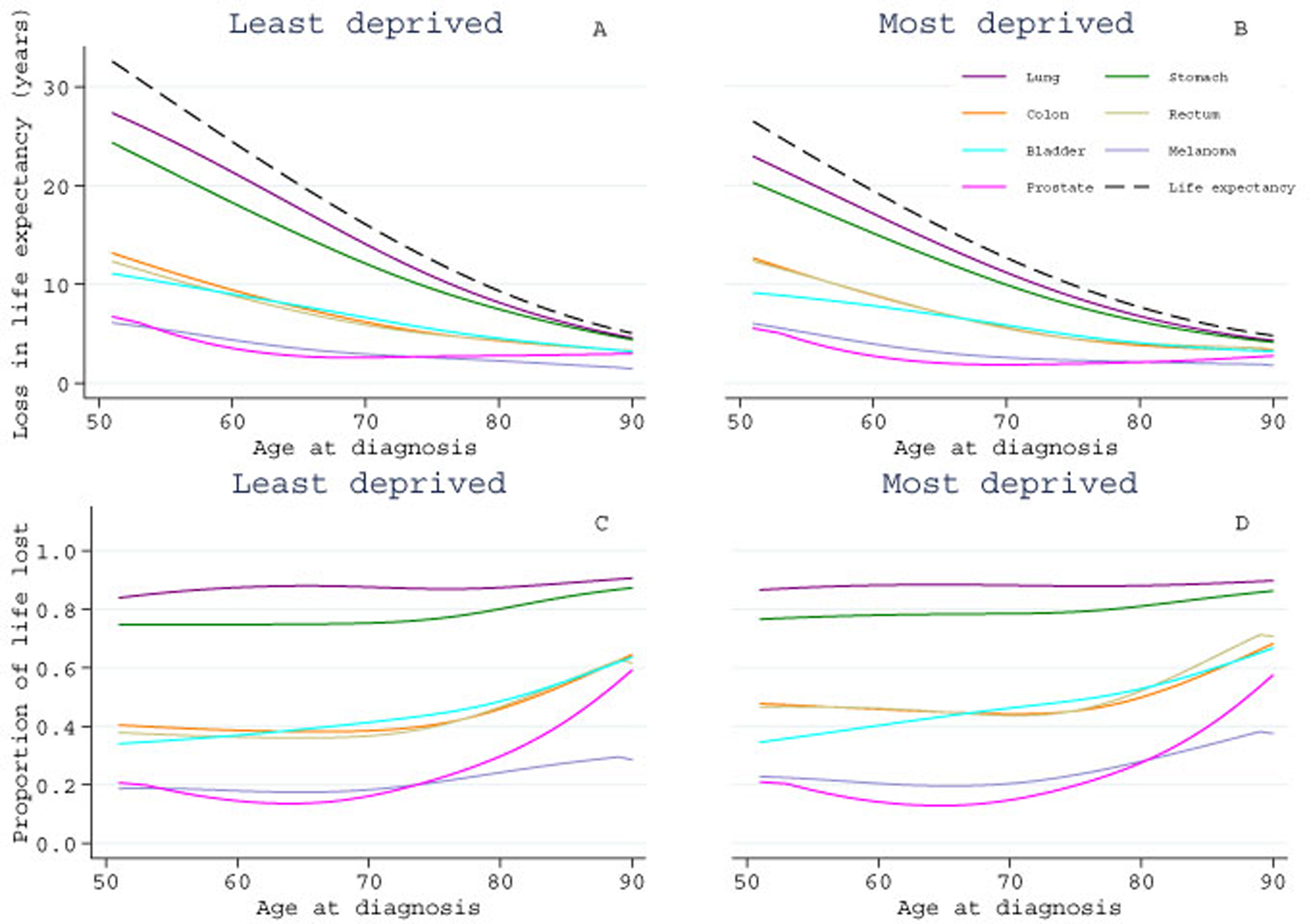

Supplement: Supplementary Figure [file bjc2017300x2.tif]
